# Polymer-Based Hydrogel Loaded with Honey in Drug Delivery System for Wound Healing Applications

**DOI:** 10.3390/polym15143085

**Published:** 2023-07-18

**Authors:** Siti Nor Najihah Yasin, Zulfahmi Said, Nadia Halib, Zulaiha A Rahman, Noor Izzati Mokhzani

**Affiliations:** Department of Basic Sciences and Oral Biology, Faculty of Dentistry, Universiti Sains Islam Malaysia, Tower B, Persiaran MPAJ, Jalan Pandan Utama, Pandan Indah, Kuala Lumpur 55100, Malaysia; sitinajihah.yasin@gmail.com (S.N.N.Y.); nadia.halib@usim.edu.my (N.H.); zulaiha@usim.edu.my (Z.A.R.); n.izzati9802@raudah.usim.edu.my (N.I.M.)

**Keywords:** wound healing, wound dressings, hydrogel, honey, natural polymer, synthetic polymer

## Abstract

Excellent wound dressings should have crucial components, including high porosity, non-toxicity, high water absorption, and the ability to retain a humid environment in the wound area and facilitate wound healing. Unfortunately, current wound dressings hamper the healing process, with poor antibacterial, anti-inflammatory, and antioxidant activity, frequent dressing changes, low biodegradability, and poor mechanical properties. Hydrogels are crosslinked polymer chains with three-dimensional (3D) networks that have been applicable as wound dressings. They could retain a humid environment on the wound site, provide a protective barrier against pathogenic infections, and provide pain relief. Hydrogel can be obtained from natural, synthetic, or hybrid polymers. Honey is a natural substance that has demonstrated several therapeutic efficacies, including anti-inflammatory, antibacterial, and antioxidant activity, which makes it beneficial for wound treatment. Honey-based hydrogel wound dressings demonstrated excellent characteristics, including good biodegradability and biocompatibility, stimulated cell proliferation and reepithelization, inhibited bacterial growth, and accelerated wound healing. This review aimed to demonstrate the potential of honey-based hydrogel in wound healing applications and complement the studies accessible regarding implementing honey-based hydrogel dressing for wound healing.

## 1. Introduction

Wounds are typically defined as damage to the skin as well as to epidermal- or dermal-layer structures. They can be categorized as acute or chronic wounds depending on their duration and the nature of the healing process [1]. Wound healing is a dynamic and sophisticated tissue regeneration process that repairs the damaged skin and other soft tissues locally or systematically. It involves four temporal stages: hemostasis, inflammation, proliferation, and remodeling [2].

Wound dressings play a significant role in offering the optimum conditions for wound healing and protecting the wounds from further damage and infection. Conventional dressings, including gauzes, plasters, and bandages, are used as primary and secondary dressings for protection against microbial infections [3]. However, these dressings absorb a high amount of moisture on the wound, and can dry and adhere to the wound surface and cause pain when removed [4]. Additionally, some of these dressings might not have antimicrobial, antioxidant, and other bioactive components [5]. Therefore, it is essential to design appropriate dressings that can be easily detached and do not cause any harm to the surface of wounds during dressing replacement [6]. Additionally, it should offer excellent antimicrobial activity, excellent mechanical properties, be able to deliver bioactive agents [7], provide a physical protective barrier, promote the deposition of the extracellular matrix (ECM), and maintain an optimal environment on the wound site, as well as promote the process of wound healing [8].

Hydrogels are three-dimensional (3D) networks made up of hydrophilic polymers formed through hydrogen or covalent crosslinking using the process of a physical or chemical reaction [9]. Hydrogels are commonly employed as wound dressings and have been proven effective, mainly for treating wounds and skin ulcers [10]. These 3D polymer networks can absorb a tremendous amount of liquid, offer a humid environment, excellent biocompatibility [11], biodegradability, and adhesion that can efficiently stimulate cell proliferation and facilitate the process of wound healing as well as improve the stage of wound repair [5]. In addition, the hydrophilic groups in the polymeric chains cause the hydrogel dressings to retain water, with a higher water content ensuring a good porosity, softness, and elasticity [12] and a cooling effect, thus minimizing pains upon removal [13].

Hydrogels can be prepared through natural polymers, including alginate, chitosan, hyaluronic acid, cellulose, starch, gelatin, etc., or synthetic polymers such as polyvinyl alcohol (PVA), polyacrylamide, polyethylene glycol (PEG), etc., or a combination of both polymers [14]. These combinations, which are known as hybrid polymers, could enhance their individual physicochemical, mechanical, and biological properties and promote healing [13]. Some examples of this combination include PVA/chitosan [15], PVA/starch [16], polyacrylamide/chitosan [17], etc. Hydrogel-based wound dressings are well recommended for their healing-promoting properties, which speed up the proliferation and epithelialization processes [8]. Therefore, they are acceptable as first aid for wound care [18]. Excellent wound dressings that are prepared from the polymers as mentioned earlier can be further enhanced by incorporating nature-based bioactive agents [19].

Honey has been applied for centuries as a treatment for infected wounds to accelerate the process of wound healing. Honey is gaining considerable attention as a regenerative agent to treat ulcers, bed sores, skin infections, wounds, and burns. It offers antimicrobial, anti-inflammatory, and antioxidant activity and maintains a moist wound environment that acts as a protective barrier to prevent infection [20]. It also demonstrates the ability to recover the development of new tissue to heal the wound through epithelization. Honey rapidly clears wounds when applied topically to promote the deep healing of wounds with infection [21]. The lower pH of honey (pH 3.5–4.0) could inhibit protease activity, which increases oxygen release from hemoglobin, and promotes macrophage and fibroblast activity on a wound site. In contrast, hydrogen peroxide sterilizes the wound and stimulates vascular endothelial growth factor (VEGF) production [22].

The application of honey-based hydrogel wound dressings has been practiced in the biomedical field. Some studies have demonstrated that honey-based hydrogel dressings can increase water absorption and swellability, support epithelization, stimulate cell proliferation, inhibit bacteria growth, and accelerate wound healing [23,24,25]. This review aimed to focus on the potential of using honey incorporated into hydrogel patch formulation as a promising approach for wound healing applications and highlight the honey-based hydrogel’s properties in treating wound infection.

## 2. The Phase of Wound Healing

**Figure 1:**
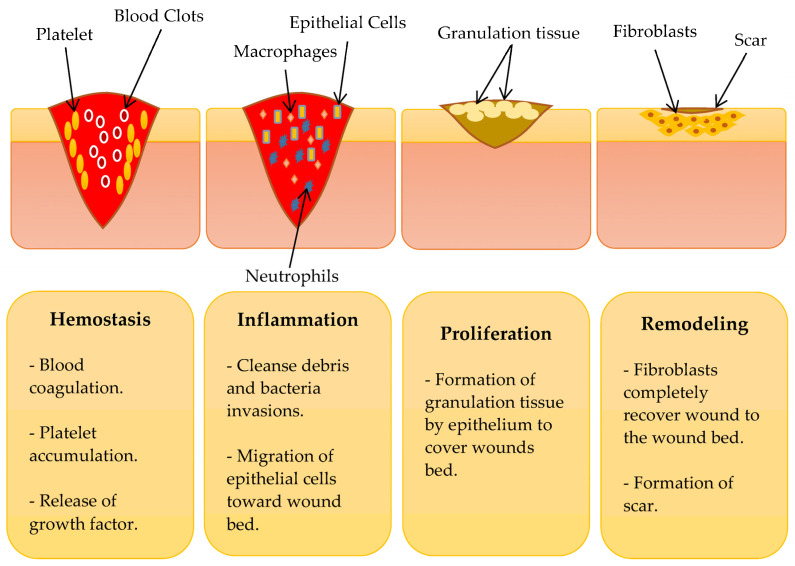
The phases of wound healing. Adapted from Ref. [33].

The process of wound healing is dynamic and sophisticated, consisting of four overlapping phases: hemostasis, inflammation, proliferation, and remodeling [26,27] (Figure 1). Hemostasis begins with blood coagulation, including the formation of fibrin clots, degranulation, platelet accumulation, and release of growth factors. As a result, fibroblasts, macrophages, and endothelial cells become involved in wound healing [28].

In the inflammatory stage, neutrophils protect the wound against pathogenic infections and cleanse the wound from cellular debris to create a favorable condition for rapid healing [29]. Exudates are responsible for inflammation symptoms, such as redness, warmth, erythema, and swelling of the damaged skin. New epithelial cells infiltrate the wound environment to replace the dead cells due to damaged skin [19]. The severity of the damage determines the duration of the hemostasis and inflammatory stages [30].

In the proliferative stage, cell proliferation and connective tissue formation occur. Next, ECM components, such as hyaluronic acid and glycosaminoglycan, contribute to the production of granulation tissue to replace the primary clot development. This stage can last several days to a few weeks following injury [31]. The final stage is a remodeling or maturation phase that starts around weeks and lasts up to months. Finally, the surface of the damaged tissue is fully recovered with fibroblast cells with a scar formation [32,33].

## 3. Classification of Wound Dressings

**Figure 2:**
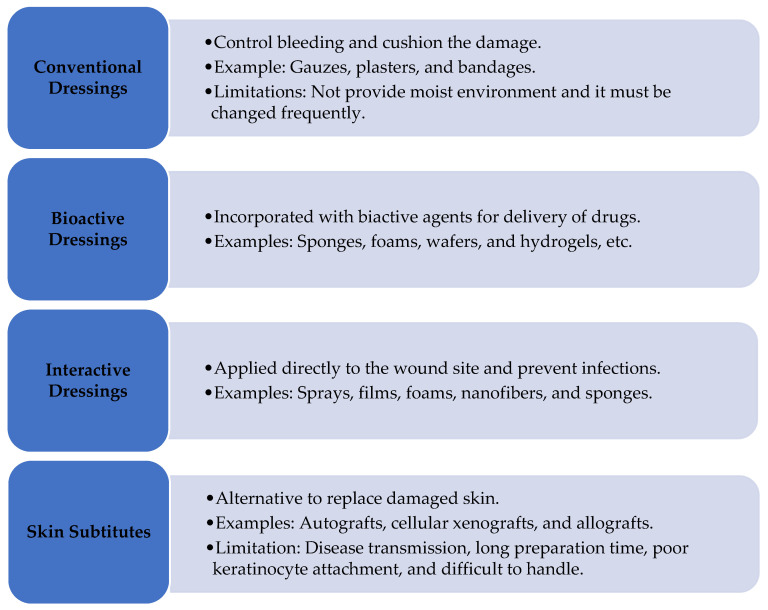
Classification of wound dressings. Adapted from Ref. [19].

Acute or chronic wounds need proper treatment to evade any shortcomings that may arise throughout the healing process. Conventional, bioactive, and interactive dressings and skin substitutes are applied to treat wounds [19] (Figure 2). Conventional wound dressings, or passive dressings, protect wounds against external substances, infection, and damage. Additionally, these dressings function to control blood, cover and absorb wounds, and cushion the damage. Examples of conventional dressings are gauze, plasters, and bandages. However, some limitations of these dressings are that they do not provide a moist environment to the wound bed [34] and must be changed frequently during the healing process, which may cause more skin damage [35].

Bioactive wound dressings are designed to provide bioactive compounds. To improve the therapeutic efficiency of these dressings, they might be incorporated with antimicrobial agents, growth factors, nutrients, nanoparticles, vitamins, plant extracts, and other natural biomaterials to the wound site to promote the healing process [36]. Some formulation-based bioactive wound dressing examples include sponges, foams, wafers, hydrogels, films, membranes, and nanofibers [37]. In addition, bioactive wound dressings have properties that include non-toxicity, excellent biocompatibility, and biodegradability [19].

Interactive dressings are applied directly to the wound site, removing debris, providing a moist environment, and preventing infections [38]. Examples of these dressings include sprays, films, foams, nanofibers, and sponges. In addition, they are favorable for re-epithelization due to excellent oxygen concentration and pH control [39].

Skin substitutes are wound dressings that are developed to restore damaged skin. They are made up of epidermal and dermal layers that are formed by fibroblasts and keratinocytes on collagen matrices. The primary mechanism of these dressings is to secrete and stimulate growth factors through which epithelization is achieved [3]. Autografts, acellular xenografts, and allografts are some forms of skin substitute wound dressings. The advantages of these wound dressings include minimizing scar formation, providing pain relief, and accelerating healing. However, some limitations of these dressings are possible disease transmission, long preparation time, poor keratinocyte attachment, difficulty handling, etc. [40].

## 4. Polymer-Based Hydrogels for Wound Healing

**Figure 3:**
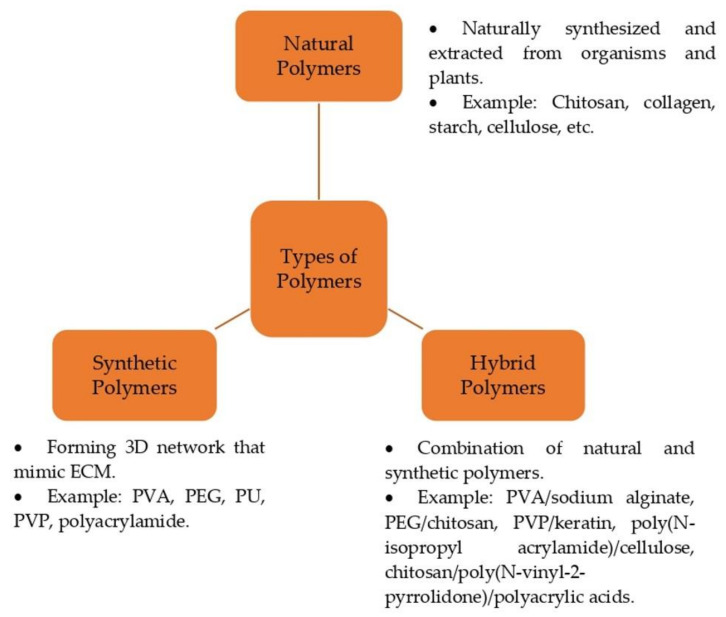
Types of polymers.

The polymer-based hydrogel can be employed in biomedical applications for wound healing, drug delivery, and tissue engineering [41]. They can be classified as natural, synthetic, and hybrid (combination of natural and synthetic) polymers (Figure 3). Excellent polymer-based hydrogel wound dressings should have appropriate features, including good biocompatibility and biodegradability, meaning the hydrogels could fully degrade after a duration [5,11,42]. Additionally, it should have adequate adhesion and excellent mechanical properties to ensure it can adhere to and cover the wounds entirely to prevent microbial infection [43]. In addition, the hydrogels should provide and maintain a humid environment at the wound site for cell migration and proliferation [11,44]. Therefore, dressing selection needs careful consideration before promoting the healing process [45].

### 4.1. Natural Polymer

Natural polymers have been applied over the centuries as the primary bioactive substance in biomedical fields [45]. These polymers are naturally synthesized and extracted from organisms and plants. Natural polymers including chitosan, collagen, starch, cellulose, sodium alginate, agarose, gelatin, and hyaluronic acid are some examples that are broadly utilized in synthesizing hydrogel wound dressings [14,46].

The interest in utilizing natural polymers as a hydrogel includes for their biodegradability, biocompatibility, non-toxicity, and low immunogenicity, and the structures are similar to that of ECM [47]. They may also produce by-products that are well tolerated by living organisms without triggering toxic reactions when subjected to enzymatic degradation [48]. However, they have some limitations, such as pathogenic contamination, uncontrollable degradation rate, complex modification, and low mechanical properties, which may restrain tissue regeneration application [49].

### 4.2. Synthetic Polymer

Synthetic polymers are beneficial in a few properties over natural polymers, such as endless forms, tunable properties, non-toxicity, and established structures [45]. Synthetic polymers are typically designed to mimic the structures of natural polymers, with minor modifications to enhance desired properties. These polymers contribute to forming a controlled 3D network with a high molecular weight, new functional groups, and charged groups. Some examples of synthetic polymers include PVA, PEG, polyurethane (PU), polyvinyl pyrrolidone (PVP), and polyacrylamide [5]. In addition, the derivations of cellulose, acrylic acid polymers, and vinyl polymers are some of the most commonly used synthetic polymers [46]. The limitations of these polymers are that they have insufficient cell adhesion sites, require chemical modifications to improve cell adhesion [50], are impermeable to drugs and proteins, and have poor mechanical stability [51].

### 4.3. Hybrid-Based Polymers

Hybrid-based polymers are developed through the combination of at least two or more polymer-based natural and synthetic polymers. Each polymer holds specific physicochemical and biological properties in a blending [52]. Several researchers have investigated if hybrid hydrogels can be widely used to overcome the limitations of both polymer types [13], as they possess the advantages of both natural and synthetic polymers in terms of their physicochemical, mechanical, and biological activities [53]. Additionally, these hybrid hydrogels could offer excellent flexibility, biocompatibility, biodegradability, and high absorption capacity and promote wound healing [54]. Some examples of hybrid polymers include PVA/sodium alginate [55], PEG/chitosan [56], PVP/keratin [57], poly(N-isopropyl acrylamide)/cellulose [58], chitosan/poly(N-vinyl-2-pyrrolidone)/polyacrylic acids [59], etc.

## 5. Physicochemical Properties and Composition of Honey

Honey is a raw substance with a sophisticated chemical composition and wound-healing properties. In addition, it has a broad range of physicochemical properties and compositions dependent upon its botanical and geographical areas [60].

The physicochemical composition of honey includes acidity, pH, moisture, ash content, hydroxymethylfurfural (HMF), sugar content, and enzyme activity. The chemical composition of honey encompasses various constituents that contribute to its biological activities. These constituents include proteins, organic acids, enzymes, phenols, flavonoids, vitamins, etc. These components play a role in honey’s beneficial effects and potential wound-healing properties [61].

A previous study has investigated the nutritional index of honey from diverse botanical areas. They found that honey’s moisture content ranges from 27–31% of honey. The ash levels were 0.15–0.9%, while the protein content was 0.2–0.8%. Additionally, the sugar content for glucose, fructose, and sucrose was 29–31%, 45–48%, and 2–4%, respectively. The HMF value also should not be more than 60 mg/kg. Its pH levels are between 3.24 to 6.1. Honey contains several active compounds, including flavonoids, organic acids, phenolic acid, vitamins, and enzymes, that may improve wound healing [62].

## 6. Biological Activity of Honey in Wound Healing

Honey has been well-known for wound treatment since ancient times. The healing properties of honey are related to its antioxidant, anti-inflammatory, and antimicrobial activities, and its capabilities of maintaining a moist wound environment, protecting the wound, and preventing pathogenic infection [20]. The immunological activity of honey is also crucial in wound healing as it has pro- and anti-inflammatory properties [63]. In addition, honey has antimicrobial properties and has the potential to counter wound infections and function as a physical barrier to the wound area, as well as promote wound healing [21]. The antimicrobial properties found in honey play a significant role in the body’s response to tissue damage [64].

Honey may aid in regenerating damaged tissues and wound healing as it contains a high sugar content, reactive oxygen species generation, and anti-inflammatory properties [65]. Additionally, honey can sterilize wound infection, stimulate the growth of tissues and re-epithelization, and reduce scar formation. These factors contribute to the four phases of wound healing, as stated above [66]. Honey demonstrates diverse effects in each stage of the wound-healing process [67]. During the inflammatory stage, honey inhibits bacterial placement, lowers pH, increases antioxidant action, increases peroxide generation, and releases pro-inflammatory cytokines [68]. It then promotes epithelization and proliferation while decreasing edema and exudate in the wound during the proliferative stage. Next, during the remodeling stage, honey helps to recover the wound and prevent scar formation [32].

**Figure 4:**
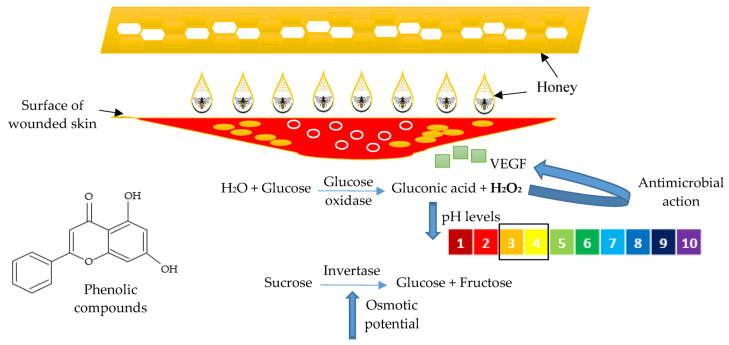
The fundamental principle of honey in wound healing. Adapted from Ref. [69].

Additionally, hydrogen peroxide (H_2_O_2_) production on glucose is another characteristic of honey that causes antimicrobial action. This compound catalyze by glucose oxidation of glucose which lead to the production of gluconic acid and hydrogen peroxide [68]. The formation of gluconic acid contributes to a decrease in pH levels, while hydrogen peroxide enhances the antimicrobial properties of honey. This cascade of events, which includes pH reduction to levels between 3.5–4.0, is crucial for initiating the tissue repair process [68,69]. Furthermore, H_2_O_2_-dependent honey may stimulate the synthesis of vascular endothelial growth factor (VEGF) and sterilize the wound site [22]. In addition to glucose oxidase, bees create invertase, which enhances the osmotic potential of honey by breaking down sucrose into fructose and glucose (Figure 4) [69]. The production of H_2_O_2_ also tends to be toxic to the cellular tissue when it is too saturated. However, it can be countered with an antioxidant compound inside the honey [70]. 

The antioxidant properties are also other medicinal properties of honey that have been studied. The antioxidant action in honey is enhanced by the presence of phenolic compounds [66]. Plants create various secondary metabolites in response to environmental stresses and oxidative damage. These compounds are transferred to honey through nectar. The phenolic compounds are divided into two categories, which are phenolic acids and flavonoids. Free radicals are scavenged by phenolic acids and flavonoids, which reduce tissue damage and inflammation. In biomedical fields, honey has been employed to treat wounds, burns, and inflammation, and has a synergistic effect when combined with antimicrobial agents [22,71]. Previous research discovered that the relative positions of OH groups in the aromatic ring affect the antioxidant effect of phenolic acids and are also found to be the most potent antioxidant among all phenolic acid compounds [72].

The current therapeutic applications used in wound management are beneficial for inhibiting bacterial infection and promoting healing [73]. Using natural products with antimicrobial properties in biomedical research has garnered considerable attention in modern medicine [71]. The excellent properties of natural products, including honey, curcumin, and aloe vera, are the most prominent arguments for applying natural products in treating wounds [74]. Honey has been a topical treatment since ancient times and has been officially recognized as a medical device in conventional medicine, and can be combined with silver dressings or other formulations [56,63]. Table 1 shows the comparison between honey and some bioactive substances that are commonly utilized in wound healing applications.

## 7. Application of Honey-Based Hydrogel for Wound Healing

**Figure 5:**
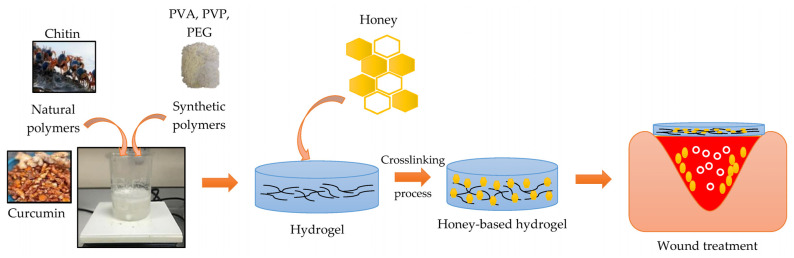
Schematic of honey-based hydrogel for wound healing. Adapted from Ref. [82].

Hydrogels combined with honey have multiple benefits and are considered ideal wound dressings to promote healing [20,78,79] (Figure 5). Hydrogels are 3D structures crosslinked with hydrophilic characteristics that can hold abundant volumes of water and other liquids [11,80]. Thus, it is applicable for wound healing due to its high porosity, excessive water content, ability to release therapeutic agents, excellent biocompatibility, biodegradability, and it can accelerate the wound healing process [5,19,81]. Furthermore, honey has been traditionally utilized as a wound dressing to accelerate and enhance the process of wound healing. Therefore, incorporating honey into the hydrogel could be effective for wound healing [20,82].

Chopra et al. prepared a natural chitosan and PVA to formulate a hydrogel film incorporating honey for wound healing treatment and evaluated their physicochemical and mechanical properties. The findings showed that the thickness and weight of the films were between 0.041 ± 0.006 to 0.055 ± 0.004 mm, and 0.425 ± 0.02 to 0.480 ± 0.04 g, respectively. The folding endurance ranged from 350 ± 15 to 445 ± 7. The folding endurance values increase as the chitosan concentration increases from F1–F5 (0.25–2%). The formulation of batch F5 (2% of chitosan) gives a smooth surface and homogenous form with little porosity, exhibiting excellent structural integrity. Additionally, the hydrogel’s moisture content increases as the chitosan concentration increases. Swelling analysis indicates that increasing the chitosan concentration may increase the water’s swelling ratio. The F5 showed the highest swelling ratio, with 300% after 24 h. For mechanical characteristics, the results showed a value from 4.74 ± 0.83 to 38.36 ± 5.39 N for tensile strength, and 30.58 ± 3.64 to 33.51 ± 2.47 mm for elongation at the break, respectively. A strong interaction and network between the polymers could enhance the mechanical characteristics of the hydrogel film. In addition, an antimicrobial study against *Staphylococcus aureus* (*S. aureus*) demonstrated that a honey-based hydrogel film exhibited antimicrobial activity with excellent bacteriostatic ability. The F4 showed an excellent antimicrobial effect, with the diameter of the inhibition zone being 5.01 ± 0.32 mm [83].

Additionally, a study by Gopal et al. incorporated Kelulut honey and Tualang honey into cellulose/PEG hydrogels to treat wound infections. The finding showed that the honey hydrogels showed excellent antimicrobial activity compared to the control hydrogels. Tualang honey hydrogels exhibited the highest zone of inhibition for negative *Escherichia coli* (*E. coli*), and *S. aureus*, which could be influenced by the highly acidic component with pH 3.55–4.0 which may inhibit both bacteria. For *E. coli*, the Kelulut honey hydrogels showed slightly higher inhibition zones than the Tualang honey hydrogels. Meanwhile, for *S. aureus*, the Tualang honey hydrogels exhibited higher inhibition zones than the Kelulut honey hydrogels. In vitro cell viability testing indicated that both honey-based hydrogels recorded the maximum cell viability (90%) compared to control hydrogels without the incorporation of honey, which recorded the minimum viability [23].

Lo et al. conducted a study that formulated cellulose/poly(lactic-co-glycolic acid) (PLGA) patches incorporated with Kelulut honey for aphthous stomatitis treatment. The ATR–FTIR study was utilized to analyze the morphology of the patches. In vitro cell viability analysis indicated that the Kelulut honey patch stimulated an incre in cell viability percentage by more than 90% compared to the control, which can promote angiogenesis by supporting tissue regeneration and skin re-epithelization. Additionally, the PLGA polymer released the honey into the extracellular matrix and rapidly closed the wound gap. In vivo analysis also demonstrated that the honey patches could inhibit the growth of *E*. *coli* in the first 2 h, followed by the inhibition of *S. aureus* in the next 2 h [84].

Zekry et al. investigated the PVA/honey hydrogel for wound healing. They prepared Manuka honey (MH)/pomegranate peel powder (PPP)/PVA (10%/1%/12%), MH/PPP/PVA (20%, 2%, 10.5%), MH/PPP/PVA (25%/2.5%/9.7%), MH/PPP/BV/PVA (25%/2.5%/0.01%/9.7%), and LH/PPP/BV/PVA (25%/2.5%/0.01%/9.7%). Scanning electron microscopy (SEM) was used to analyze the morphological structures of all formulations. The in vitro release study displayed that the honey was released over 24 h with a low adhesion to the wound site, stimulating cell proliferation and re-epithelization. Additionally, in vivo analysis of the wound healing activity indicated that all treated groups achieved complete healing on day 10, compared to the PVA control group (day 13) and no treatment groups (day 14) which demonstrated slowed healing processes. Moreover, at day 3 and 5, the commercial Medihoney^®^ group showed a higher percentage of wound closure compared to the PVA control and no treatment groups. Additionally, the honey hydrogel inhibited 90–98% of the *S. aureus* and *E. coli* growth, which showed good antimicrobial activity compared to controls [85].

Samraj et al. studied a combination of Kelulut honey with curcumin in the nanofibrous composite hydrogel membrane to treat wound healing. The findings showed that the impregnation of curcumin and honey promotes healing by stimulating cell migration and promoting recovery through anti-inflammatory properties. In addition, impregnating honey with curcumin promotes new cell regeneration and prevents scar formation. In vitro and in vivo rat models showed improved recovery and no cytotoxicity compared to control groups without treatment. Furthermore, antioxidant and antimicrobial studies demonstrated that the activity of wound healing with the hydrogel membrane was significantly higher than curcumin and honey alone. Therefore, incorporating honey into composite hydrogel membranes may assist in wound healing [86].

A previous study by Noori et al. developed a nanocomposite hydrogel using PVA/chitosan/honey/montmorillonite (PCMH). SEM and XRD were employed to perform the morphological analysis of the hydrogel film. Additionally, swelling tests were performed at 37 °C, and the results demonstrated that the swelling increased as the temperature increased. Furthermore, the 3-(4,5-dimethylthiazol-2)-2,5-diphenyltetrazolium bromide (MTT) analysis revealed that the PCMH hydrogel had a higher cell viability above 75% after 24 h, indicating no cytotoxicity. For pure chitosan, it was shown that the cell viability was more than the control group. This indicated that the pure chitosan itself could stimulate cell proliferation. An in vitro study against *S. aureus* has shown that PCMH hydrogel showed a more significant antibacterial value of higher than 99%, which demonstrated that it can restrict the growth of bacteria. Additionally, wound healing activity was evaluated in rats through in vivo analysis, and the results showed that PCMH hydrogel reduced the wound area more significantly than the control group and showed better wound healing ability, a rapid rate of honey release, restricted bacterial growth, and reduced the length of the wound healing process through cell reepithelization and proliferation. These results indicate that honey-based hydrogels could be applied as a wound-healing treatment [24].

The studies in vitro and in vivo performed by El-Kased have incorporated Egyptian honey (25, 50, and 75%) into chitosan/polyacrylic acid hydrogels for treating burn-wound healing. The findings showed that all hydrogel formulations exhibited a rapid swelling behavior due to their porous structure, providing a large surface area for rapid solvent uptake. Additionally, the swelling index was found to be inversely proportional to the honey concentration, indicating that an increase in honey concentration results in a decrease in the hydrogel’s swelling percentage. This factor may be affected by the polymer’s viscosity, which can impact the swelling process. In vitro release studies revealed that the release of honey from the hydrogel depended upon the honey concentration. Among all formulations, hydrogels with the lowest concentration of honey (25%) showed superior sustained release with 70% of release over 3 h. In vitro antimicrobial analysis showed that 75% of honey incorporated into hydrogels showed the highest healing rate as it stimulated cell re-epithelization and excellent antimicrobial activity compared to pure honey and commercial products [87].

Yang et al. developed nanofibrous silk fibroin and polyethylene oxide (PEO) with various concentrations of Manuka honey (10%, 30%, 50%, and 70% *w*/*v*) using an electrospinning technique. The FTIR was used to study the structural behavior of the fibrous matrices. The findings showed that the honey-based hydrogel dressings exhibited antimicrobial activity against *E. coli*, *S. aureus*, *P. aeruginosa*, and MRSA, in which the results revealed that the non-honey dressing was approximately zero, but antimicrobial activity improved to around a 50%, 28%, 57% and 40% inhibition of *E. coli*, *S. aureus*, *P. aeruginosa*, and MRSA, respectively, for the 70% *w*/*v* honey hydrogel over 24 h. Furthermore, in vitro biocompatibility analysis showed that hydrogel containing honey had a higher viability than the control. However, the increasing concentration of honey did not change the cell viability, demonstrating that the incorporation of honey does not negatively affect the excellent biocompatibility of the hydrogel. Additionally, in vivo analysis in a rat dorsal wound model showed that the wounds treated with 70% honey hydrogel wholly recovered, whereas both the control group and commercial Aquacel^®^Ag wound dressing group had only slight reductions in wound size [88].

Another study by Tavakoli and Tang fabricated a polyvinyl alcohol/Manuka honey hybrid hydrogel wound dressing with borax as a crosslinking agent. Hydrogels prepared with 1% borax demonstrated adequate biocompatibility, a sustained release of honey in the ulcer bed, and no burst release of antibiotics. The addition of borax also increased the mechanical durability of the honey/PVA hybrid and prevented hydrogel degradation during the swelling process. This thin layer of hydrophilic gel may improve the wound-healing process and reduce the risk of contamination. The results demonstrated that the honey showed good antibacterial activity against *S. aureus* in all samples, especially in the samples with a 1% crosslinking agent. The results showed that the PVA/borax/honey hybrid hydrogel demonstrated the greatest swellability and stability and had excellent antimicrobial activity, and indicated that PVA/honey hydrogel produced the best characteristics for applying to wound dressing [25].

Durai and Sizing fabricated chitosan hydrogel films impregnated with 8% Manuka honey to treat wounds. The results revealed that honey increased the folding endurance, with the honey hydrogel films surviving a mean of 289 folds compared to 143 folds for the non-honey films. This result demonstrates a greater flexibility of the honey hydrogel film due to the hygroscopic effect of honey. Additionally, honey reduced the swelling ratios of the hydrogel films and inhibited the growth of *S. aureus* and *E. coli*. In an in vivo analysis of a rat dorsal wound model, the honey hydrogel showed an increased wound gap compared with control groups of non-honey and ointment. The honey hydrogel and non-honey hydrogel showed closures of 94% and 78% after 12 days of treatment, compared with the ointment-treated group and the non-treated control whose wounds showed closures of 86% and 64%, respectively [89].

Zohdi et al. developed a hydrogel dressing incorporating Gelam honey into the polyvinyl pyrrolidone (PVP)/protein-free agar/polyethylene glycol (PEG) hydrogel with a 6%, 8%, 10%, and 15% concentration of honey. The finding showed that the honey hydrogel and the control group had good uniformity and transparency with a 3–4 mm thickness. Additionally, the pH of the honey hydrogel was slightly acidic, with a value of pH 4.3, while the control group had a pH of 5.3. This slight acidity in the hydrogel may be due to the natural acidic properties of honey, which typically has a pH ranging from 3.2 to 4.5. For swelling analysis, the honey hydrogel demonstrated a high capability in absorbing fluid compared to the control group. The in vivo analysis in rats revealed that the honey hydrogel dressing stimulated wound closure and promoted the process of reepithelization better than the control group. Furthermore, the histopathological analysis showed that the honey hydrogel attenuated the inflammatory response on day 7, earlier than the control group. Moreover, honey hydrogel facilitates the growth of granulation tissue and blood capillary and collagen synthesis, which is effected by the generation of hydrogen peroxide by honey [90].

Khoo et al. compared a Tualang honey wound dressing and hydrofiber silver-treated wound dressing. The results demonstrated that the Tualang honey dressing had more flexibility, less adherence, easily peeled, and caused less fluid accumulation in the wound site. Furthermore, according to an in vivo study, using Tualang honey for dressing burn wounds resulted in significantly greater wound contraction than applying hydrofiber silver dressing. Furthermore, on day 6, the wound area became smaller and showed increasing cell epithelization. Additionally, the Tualang honey -treated wound dressing showed a lower bacterial growth of *Pseudomonas aeruginosa*-inoculated wounds and excellent antibacterial activity [91].

## 8. Cell Migration and Proliferation on Honey-Based Wound Dressings

**Figure 6:**
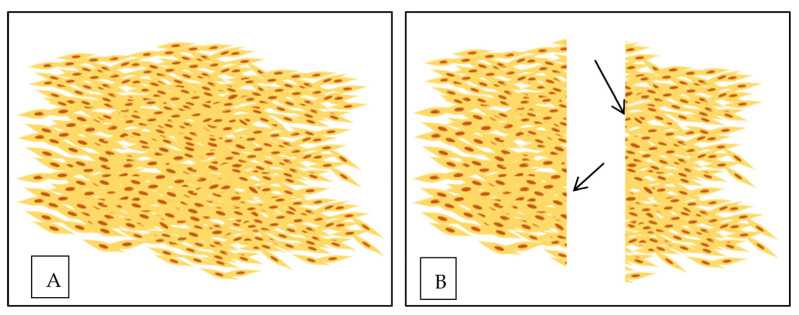
Illustration of in vitro wound healing assay. (**A**) Fibroblast cells form a confluent monolayer. (**B**) In vitro “wound” was created by a straight line scratch across the fibroblast monolayer [94].

The scratch- or wound-healing assay is a cost-effective and straightforward experimental method for investigating cell migration [92]. The assay involves growing a cell monolayer in a multiwell assay plate, creating a “wound” or scratch, and then capturing images at regular intervals to measure and quantify cell migration [93] as shown in Figure 6. Scratch assays are commonly employed to study the molecular mechanisms that influence cell migration and to identify therapeutic compounds that can modulate cell migration for potential treatments. Therefore, it is crucial to develop reliable methods for quantifying and comparing migration rates of different scratch assays to advance biomedical research [94]. The wound closure percentage was calculated using the following formula:

$$\%\;\mathrm{Wound}\;\mathrm{Closure}=\frac{A_{0}-A_{T}}{A_{0}}\times 100\%$$

where A_0_ is the wound area measured after scratching, and A_T_ is the area of the wound measured at a predetermined time.

There are limited studies on utilizing cell-culture applications to perform cellular migration upon honey-based dermal wound dressings, as there are broad studies that have carried out the application of honey dressing in animals to study the effectiveness of honey in wound healing. However, several studies utilize pure honey (with a dilution factor) for wound healing analysis.

For instance, Chaudhary et al. studied the cell migration assay under 0.1% of Manuka honey and 0.1% Jamun honey on primary fibroblast cells from a neuron differentiation medium (NDM) and a decalcified bone matrix (DBM) skin. The results showed that both kinds of honey could stimulate cell proliferation against fibroblast cells over 24 h. However, DBM cells with Manuka honey and Jamun honey migrated faster than NDM cells at 24 h [95].

Ranzato et al. performed a scratch-wound assay on the fibroblast cells using 0.1% *v*/*v* Manuka, buckwheat, acacia honey, and platelet lysate (PL). The finding showed that the cells exposed to buckwheat and acacia honey showed a higher rate of wound closure at 24 h compared to controls, while Manuka honey showed a lower effect against fibroblast cells [96].

The study by Ebadi and Fazeli performed a wound healing analysis on human dermal fibroblasts using honey and an ethanol extract of propolis (EEP). The finding showed that 100 μg/mL and 200 μg/mL concentrations of EEP demonstrated the highest percentages of wound closure compared to the control and DMSO control. After 48 h, the wound healed entirely at the 100 μg/mL and 200 μg/mL concentrations. For the honey analysis, the 25 μg/mL to 200 μg/mL concentrations showed a slight increase in the percentage of wound closure, while for the 100 μg/mL and 200 μg/mL concentrations, the wound healed after 48 h, faster than both control groups. The EEP and honey concentrations of 100 μg/mL and 200 μg/mL showed remarkable wound closure after 24 and 48 h compared to both control groups [97].

An MTT assay also can be performed to assess cell proliferation. A study by Lau et al. performed a cell proliferation assay under different concentrations of Tualang honey on human periodontal ligament fibroblast cells (HPDLF). The finding showed that 0.02% Tualang honey concentration stimulated a higher proliferation rate than the control. However, at a higher concentration of Tualang honey (5%), the cells became rounded and floating, indicating that a higher honey concentration could inhibit cell proliferation [98].

Additionally, Shamloo et al. studied the cell proliferation and biocompatibility of human fibroblast cells using various concentrations (0, 5, 10, and 20%) of a chitosan/honey hydrogel. The finding demonstrated that a 10% concentration of chitosan/honey hydrogel stimulated the highest cell proliferation compared to other hydrogels. It also demonstrated that the addition of honey into hydrogel could offer maximum nutrients for cells, which may increase cell proliferation, as well as cell viability [99].

A study by Sarhan et al. analyzed the cell proliferation of human fibroblast cells when using various types of honey hydrogel (0, 25, 50, 75, and 100% extraction). The findings showed that 100% honey extraction stimulated the highest cell proliferation (>90%) compared to other hydrogels and the positive control, commercial Aquacel^®^Ag. In this study, the Aquacel^®^Ag showed cytotoxic signs with a cell viability of 9% [100].

## 9. Toxicological Information of Honey-Based Wound Dressings

It is essential to consider the toxicological aspects associated with honey-based wound dressings [101]. Among many types of toxicological analyses, the MTT assay is a widely utilized method to evaluate cell viability and cytotoxicity in vitro, which makes it suitable for toxicological analysis in wound-healing applications [102]. Table 2 shows in vitro MTT assays related to applying honey-based wound dressings.

## 10. Regulatory Information of Honey-Based Wound Dressing

Honey-based wound dressings are classified as medical devices. They are regulated by various regulatory agencies worldwide [66], including the US Food and Drug Administration (FDA) [105], European Medicines Agency (EMA) [106], National Medical Products Administration (NMPA) [107], Therapeutic Goods Administration (TGA) [108], Health Sciences Authority (HSA) [109], and Medical Device Authority (MDA) [110]. It should be noted that the regulatory requirements for honey-based wound dressings may vary depending on the country and region in which they are commercialized [66].

The importance of providing the regulation information before they can be sold in a country is to assure the quality, efficacy, and safety of products that are used for wound care [111]. Regulatory bodies set standards and guidelines for manufacturing, labeling, and marketing wound-care products, including honey-based wound dressings, to ensure that they meet specific criteria and do not harm patients [112]. By adhering to these regulations, manufacturers can ensure that their products are effective and safe for use, and healthcare providers and patients can have confidence in their products [113]. Additionally, regulatory information can help healthcare providers. Patients make informed decisions about wound care products based on their specific needs and circumstances [111,114]. Table 3 describes the regulatory requirements for honey-based wound dressings based on the country.

## 11. Patent Information on Honey-Based Wound Dressings

Patent information in wound dressing refers to the documentation of a novel invention or discovery related to wound dressings, registered with the appropriate government agency for exclusive rights of use and distribution by the inventor or assignee for a certain period [115]. This information can include detailed descriptions of the wound dressing composition, manufacturing methods, potential applications, and any relevant testing or clinical trial results [116].

The importance of patent information in wound dressing lies in the potential value it can offer to researchers, manufacturers, and clinicians involved in wound care. By studying patented wound dressings, researchers can gain insights into new materials and technologies that may improve the efficacy and safety of wound healing [117]. In addition, manufacturers can use this information to develop and market innovative wound dressings that offer unique benefits to patients. Moreover, clinicians can stay informed about new wound dressing options that may help their patients heal faster and with fewer complications [118]. Overall, patent information in wound dressing is an essential resource for anyone involved in wound-care research, development, and clinical practice, providing insights into new technologies and innovations that may help improve patient outcomes and advance the field of wound healing [117,118,119]. Table 4 describes patent information for honey-based wound dressings.

## 12. Commercialized Product of Honey-Based Wound Dressings

Commercializing a honey-based wound dressing involves bringing the product to market and selling it to healthcare providers, medical facilities, and end-users [129]. Table 5 shows some recent honey-based wound dressings commercialized in the market. These commercialized honey-based wound dressings effectively manage and treat various wounds, including burns, diabetic ulcers, surgical wounds, pressure ulcers, etc. [25,130]. They are also known for reducing inflammation and promoting faster healing compared to traditional wound dressings. However, they should be used under the guidance of a healthcare professional before independent application of these dressings [1,131].

## 13. Conclusions and Future Perspectives

Wound healing is a sophisticated process that involves the replacement of damaged tissue layers and cellular structures. Numerous approaches have focused on wound-care management, including developing new therapeutic approaches and technologies for wound management. Hydrogel wound dressings have gained attention among researchers due to their rapid wound healing properties and their ability to offer a moist environment, good biodegradability, and protection against bacterial infections. Improving the physicochemical, mechanical, and biological properties, and the wound-healing ability of hydrogel materials, is the primary goal when developing hydrogels, mainly achieved through blending natural and synthetic polymers with the addition of other bioactive substances, such as honey, which is beneficial for wound healing. The addition of honey during in vivo and in vitro studies into formulated hydrogel wound dressings has been found to prevent bacterial infections, enhance their absorption capacity, and accelerate wound healing, due to its anti-inflammatory, antimicrobial, and antioxidant activities. Moreover, the blending of polymers could be enhanced by incorporating other additives, such as cross-linkers, to enhance their mechanical properties, flexibility, biocompatibility, biodegradability, high absorption, etc. Although there are extensive in vivo and in vitro analyses that have shown efficacy in wound healing, its implementation in clinical fields still needs to be managed to ensure the safety and effectiveness of polymer-based hydrogel formulations in human applications.

## Figures and Tables

**Table 1:** Comparison between honey and other bioactive substances for wound healing applications.

Bioactive Substances	Advantages	Disadvantages	References
Honey	- Rapid epithelization and wound contraction. - Reduced pain and inflammation. - Cost-effective.	- Give a stinging sensation in some patients that can cause some discomfort.	[20]
Epidermal Growth Factor	- Maintains tissue hemostasis by regulating cell survival, proliferation, migration, and differentiation.	- Short in vivo half-life due to low stability. - Restricted absorption to the wound site. - Elimination by exudation before reaching the wound site. - High-cost dressings.	[75]
ECM Protein	- Regulates cell differentiation, migration, and proliferation.	- Uncontrolled cell growth and invasion. - Impaired apoptosis and cell differentiation. - Dysfunction of all the normal functions of the skin.	[76]
Silver sulfadiazine	- Prevent and treat wound infection (second and third-degree burns).	- Toxic to fibroblasts when applied in high concentrations.	[77]

**Table 2:** In vitro MTT assay of honey-based hydrogel wound dressings.

Type of Formulation	Formulation Matrices	Percentage of Honey in the Formulation	Site of Application	Findings	References
Cellulose hydrogel	Sodium carbomethyl cellulose (SCMC)/hydroxypropyl methyl cellulose (HPMC)/polyethylene glycol (PEG)/honey	Kelulut honey (22%) Tualang honey (22%) Asian honey (33%)	Human skin fibroblast cells	All samples showed higher cell viability (<90–100%) compared to control group. Kelulut honey > Asian honey > Tualang honey.	[23]
Hybrid hydrogel film	6% *w*/*w* PVA/3, 6, 10% *w*/*w* borax/honey	5 g honey	Human fibroblast cells	All samples with different borax concentrations showed <90% cell viability compared to control group.	[25]
Hybrid hydrogel	Cellulose/poly(lactic-co-glycolic acid)(PLGA)/Kelulut honey	Not stated	Human skin fibroblast cell	Honey hydrogel showed maximum cell viability with 218.35 ± 7.80% compared to control.	[84]
Nanofibrous hydrogel	Pomegranate/PVA/honey	Manuka honey: 25% Bee venom honey: 0.01% Lyophilized multiflora honey: 25%	L929 mouse fibroblast cells	All hydrogel scaffolds with different concentrations (and different types of honey) showed <100% cell viability compared to control group, which indicates that all hydrogels have no cytotoxicity against skin cells. Promotes cell migration and proliferation.	[85]
Hybrid hydrogel	3% *v*/*v* chitosan/5% *w*/*v* gelatin/10% *w*/*v* PVA/Iran honey	0, 5, 10, and 20% *v*/*v* honey	Human fibroblast cells	Chitosan-based hydrogel showed non-toxicity impacts on the cells, and showed highest biocompatibility. It demonstrated that the addition of honey-based hydrogel could offer the cells with nutrients and increase cell proliferation.	[99]
Electrospun nanofibrous hydrogel	- 3.5% *w*/*v* chitosan/7% *w*/*v* PVA/30% honey - honey/PVA/chitosan (HPCS) - Honey/PVA/chitosan/*Cleome droserifolia* (HPCS-CE) - honey/PVA/chitosan/*Allium sativum* (HPCS-AE) - HPCS/AE/CE	30% *w*/*v*(25, 50, 75, 100% extraction)	HFD4 human fibroblast cells	- HPCS and HPCS-CE showed 90% and 87% of cell viability in the 100% extract solution compared to control and commercial Aquacel^®^Ag. - HPCS-AE and HPCS-AE/CE showed decreased cell viability (68% and 75%) in the 100% extract solution.	[100]
Electrospun nanofibrous hydrogel	- 0.8% *w*/*v* sodium alginate/7.2% *w*/*v* PVA/acacia honey	0, 5, 10, 15, and 20% *v*/*v* honey	NIH3T3 fibroblast cells	- The nanofibrous hydrogel loaded with 10% honey showed the highest cell viability with 102.71 ± 1.31%. However, at 20% honey, cell viability decreased to 96.42 ± 0.93%.	[103]
Electrospun nanofibrous hydrogel sheet	Poly(ε-caprolactone)(PCL)/Manuka honey	1%, 5%, 10%, and 20% *v*/*v*	Fibroblast cells	Sample with 20% honey showed the highest cell viability compared to other group and control group.	[104]

**Table 3:** Description of the regulatory body in different countries.

Country Name	Regulatory Body	Regulatory Guidelines	Classification of Wound Dressings	Regulatory Requirements	References
United States	USFDA	21 CFR Part 820	Class I: low to moderate risk Class II: moderate to high risk Class III: high risk	Premarket approval or 510(k) application is required.	[105]
Europe	EMA	Council Directive 93/42/EEC	Class I: low risk Class IIa & IIb: medium risk Class III: high risk	Quality Management Systems (QMS) approval is required.	[106]
China	NMPA	Medical Devices Act	Class I: low to moderate risk Class II: moderate to high risk Class III: high risk	Application form is required, and need an approval before marketing.	[107]
Australia	TGA	Australian Therapeutic Goods Regulations	Class I: low risk Class II: medium risk	EU approval and CE markage is required.	[108]
Singapore	HSA	Health Product Act	Class A: Low-risk Class B: Moderate-risk Class C: High-risk Class D: In vitro diagnostic (IVD) medical devices	Approval is required.	[109]
Malaysia	MDA	Medical Devices Act 2012 (Act 737)	Class A: Low-risk Class B: Low to moderate-risk Class C: Moderate to high-risk Class D: High-risk	Conformity Assessment Body (CAB) approval is required.	[110]

**Table 4:** Patent information of honey-based wound dressings.

Type of Patents	Inventor(s)	Issued	Assignee	Descriptions	References
US7714183B2 Use of honey in dressings	Phillip Roy Caskey	11 May 2010	Derma Science Inc	- The patent application describes a flexible dressing designed for direct application to wounds to absorb exudates. - The dressing consists of an alginate fiber sheet that is fully impregnated with honey, and transforms into a gel-like state as it absorbs exudate. - The combination of the alginate fiber sheet and honey provides benefits such as moisture retention, antimicrobial properties, and wound-healing promotion.	[120]
WO2002087644A1 Wound dressings comprising a carboxymethyl cellulose fabric impregnated with honey	James William Edmonds	7 November 2002	Not listed	The patent describes the wound dressings comprising honey as also containing carboxymethyl cellulose filaments in amounts up to 50% of the weight of the honey, preferably in the form of a fabric.	[121]
US5980875A Honey preparations	Mahmoud A. Mousa	11 November 1999	Not listed	- The patent describes the methods and preparations designed to address challenges related to the local application of honey for therapeutic, cosmetic, and nutritional purposes. - These preparations consist of active ingredients derived from honey and a base containing components such as oils, gelling agents, emulsifiers, or combinations thereof. - The active ingredients found in honey, such as vitamins, sugars, enzymes, hormones, amino acids, and minerals, can be extracted from honey or other natural sources or be synthesized.	[122]
US9107974B2 Honey impregnated composite dressing having super-absorbency and an intelligent management of wound exudate, and methods of making the same	Howard Kenneth Payne, Gregory Frank Devenish	18 August 2015	Links Medical Products Inc	- The patent relates to a specialized wound dressing called a super-absorbent, honey-dosed foam/fiber composite with a gap pattern. - This dressing consists of a structured composite material made of foam and fiber, which is patterned with gaps on one side while the other side lacks such gaps.	[123]
AU2006272366B2 Therapeutic honey and method of producing same	Peter Taylor	22 September 2011	Honey Research & Development Pty Ltd.	- The patent relates to a specific type of honey characterized by the following attributes: (i) It exhibits antimicrobial activity that is not derived from peroxide; and (ii) It is derived from Leptospermum sub-tenue. - The honey undergoes a storage process for a certain duration and under specific conditions necessary to enhance its non-peroxide-based antimicrobial activity.	[124]
US10500235B2 Wound healing compositions comprising buckwheat honey and methylglyoxal and methods of use	Mark R. Wardell	10 December 2019	San Melix Laboratories Inc, Sanmelix Laboratories Inc	- The patent application presents compositions based on medicinal honey that possess broad-spectrum antibacterial properties attributed to the presence of peroxide, polyphenols, and methylglyoxal. - The application describes methods of treating wounds by directly applying the aforementioned composition or utilizing wound dressings that incorporate the composition.	[125]
WO2007045931A2 Compositions and dressings for the treatment of wounds	Stephen Cotton	26 April 2007	Not listed	- The patent describes the composition of honey ranging from 30% to 99.5% *w/w,* and a naturally occurring plant extract with antibacterial properties ranging from 0.5% to 15% *w*/*w* as having proven effectiveness in wound treatment. - These compositions can be directly applied to wounds or can be applied to a flexible material, either as a coating or impregnation.	[126]
US6956144B2 Honey-based wound dressing	Peter Molan	18 October 2005	ApiMed Medical Honey Ltd.	- The patent application describes the utilization of honey in medical dressings. It involves the modification of honey by incorporating a viscosity increasing agent, which enables the creation of various compositions such as ointments, salves, and self-adhesive gels for mouth ulcers and pustules, as well as pliable or flexible sheets suitable for wound coverings. - The invention emphasizes the use of selected honeys that possess antibacterial properties beyond those attributed solely to osmolarity and sugar concentration effects.	[127]
AU2007100007A4 Improvements in and Relating to the use of Honey in Dressings	Phillip Roy, Caskey, Mardi Lewis	1 February 2007	ApiMed Medical Honey Ltd.	- This invention proposes utilizing honey with desirable qualities and viscosity in combination with various therapeutic or suitable medical dressings. - Example: Honey-impregnated dressings are expected to have significant potential in the treatment of chronic wounds, whether infected or non-infected, especially in cases where moist wound care is required.	[128]

**Table 5:** Product commercialization of honey-based wound dressings.

Type of Dressing	Examples/Products	Intended Usage	References
Hydrocolloid Dressing	MediHoney^®^	- Chronic (diabetic ulcers, venous ulcers, pressure ulcers) and acute wounds (surgical wounds) and burns. - Promotes wound healing by reducing healing time and promoting tissue growth.	[132]
Film Dressing	TheraHoney^®^	- Diabetic ulcers, venous ulcers, pressure ulcers, surgical wounds, traumatic wounds, and burns. - Stimulates wound healing by providing a moist environment, reducing pain and inflammation, and providing antimicrobial activity.	[133]
Foam Dressing	Actilite^®^	- Pressure ulcers, leg ulcers, diabetic ulcers, surgical wounds, traumatic wounds, and burns. - Protects wounds from antimicrobial activity, and provides a moist wound environment.	[134]
Alginate Dressing	Algivon^®^	- Leg ulcers, pressure ulcers, diabetic foot ulcers, and surgical wounds. - Provides antimicrobial properties, helps to absorb exudate, and promotes healing.	[135]
Mesh Dressing	Activon^®^	- Diabetic foot ulcers, pressure ulcers, venous leg ulcers, surgical wounds, traumatic wounds, and burns. - Promotes healing by providing a moist wound environment, managing exudate, reducing inflammation, and preventing infection.	[136]

## Data Availability

Not applicable.
